# Water Quality Criteria for Copper Based on the BLM Approach in the Freshwater in China

**DOI:** 10.1371/journal.pone.0170105

**Published:** 2017-02-06

**Authors:** Yahui Zhang, Wenchao Zang, Lumei Qin, Lei Zheng, Ying Cao, Zhenguang Yan, Xianliang Yi, Honghu Zeng, Zhengtao Liu

**Affiliations:** 1 State Key Laboratory for Environmental Criteria and Risk Assessment, State Environmental Protection Key Laboratory of Ecological Effects and Risk Assessment of Chemicals, Chinese Research Academy of Environmental Sciences, Chaoyang Distinct, Beijing, People’s Republic of China; 2 Solid Waste and Chemicals Management Center, MEP, Chaoyang District, Beijing, People’s Republic of China; 3 Guangxi Polytechnic of Construction, Xixiangtang District, Nanning, Guangxi Province, People’s Republic of China; 4 College of Environmental Science and Engineering, Guilin University of Technology, Guilin, Guangxi Province, People’s Republic of China; 5 Dalian University of Technology, Dalian, Liaoning Province, People’s Republic of China; Jinling Institute of Technology, CHINA

## Abstract

The bioavailability and toxicity of metals to aquatic organisms are highly dependent on water quality parameters in freshwaters. The biotic ligand model (BLM) for copper is an approach to generate the water quality criteria (WQC) with water chemistry in the ambient environment. However, few studies were carried out on the WQCs for copper based on the BLM approach in China. In the present study, the toxicity for copper to native Chinese aquatic organisms was conducted and the published toxicity data with water quality parameters to Chinese aquatic species were collected to derive the WQCs for copper by the BLM approach. The BLM-based WQCs (the criterion maximum criteria (CMC) and the criterion continuous concentration (CCC)) for copper in the freshwater for the nation and in the Taihu Lake were obtained. The CMC and CCC values for copper in China were derived to be 1.391 μg/L and 0.495 μg/L, respectively, and the CMC and CCC in the Taihu Lake were 32.194 μg/L and 9.697 μg/L. The high concentration of dissolved organic carbon might be a main reason which resulted in the higher WQC values in the Taihu Lake. The WQC of copper in the freshwater would provide a scientific foundation for water quality standards and the environment risk assessment in China.

## Introduction

Metals have become widespread contaminants in surface water due to anthropogenic discharge. Increased environmental exposure of waterborne metals has resulted in potential risks to aquatic biota [1, 2]. Copper (Cu) is a ubiquitous pollutant in freshwaters [3] and listed as one of priority pollutants in China [4]. The bioavailability and toxicity of Cu to organisms is highly dependent on the chemical speciation [5–7] which is affected by water quality parameters.The increase of the concentrations of the cations (Ca^2+^, Mg^2+^, Na^+^ and H^+^) [8, 9] and dissolved organic matter (DOM)[10–14] would lead to decrease on the toxicity for Cu to aquatic organisms. Biotic ligand model (BLM) is a mechanistic model which quantitatively assess the metal toxicity of the free metal ion by competition with the cations (e.g., Ca^2+^, Mg^2+^, Na^+^, K^+^, H^+^) and complexation by the abiotic ligands (e.g., chloride, carbonates, sulfide and DOM) in aquatic system [8, 15].The BLM has been developed to enable modeling both acute and chronic toxicity of waterborne Cu to aquatic organisms as a function of a variety of water quality characteristics[14, 16–18]. The studies on the toxicity of Cu to fish [17, 19, 20] and daphnid [8, 16, 21] species were most investigated in the acute aquatic BLMs, also to the other aquatic organisms such as crustaceans [22] and green algae [23]. Chronic BLMs has been developed to predict the toxicity for Cu to fish in relatively few study so far [24]. The Cu-BLM is therefore employed as an alternative approach to the hardness-adjusted equation and the water-effect ratio method to establish the water quality criteria (WQC) for protecting the aquatic life for Cu by the US EPA[25].

In China, current Environmental Quality Standard for Surface Water (GB3838-2002) and risk assessment for Cu are still based on the total Cu concentrations, which might not well evaluate the bioavailability and toxicity of Cu in the freshwater biota. Thus, China has embarked to develop the WQC system[26] and employ the BLM to revise the water quality standards of metals[27]. However, the WQCs for protection of aquatic organisms of Cu have not developed in China because the influence of the water chemistry on the toxicity of waterborne Cu to native species has been taken into account in few studies[28–30]. In the present study, both acute and chronic toxicity of Cu to native freshwater aquatic organisms in China were determined with water quality parameters in the tests and the toxicity data in the published literatures were collected and evaluated to calculate the WQC for the protection aquatic life criterion for Cu based on the BLM approach. The criterion maximum criteria (CMC) and the criterion continuous concentration (CCC) based on the BLM for copper in China[31].The Taihu Lake is the main drinking water sources of surrounding cities and towns as the third largest fresh water lake in eastern China [32]. The pollution of metals has been concerned due to the wastewater of agriculture and industry releasing into the lake [33]. The BLM-based WQC for Cu in the Taihu Lake was also derived using the toxicity data from native aquatic organisms. The study would provide a scientific foundation for water quality standards and risk assessment for Cu in aquatic ecosystem in China.

## Methods and materials

### Ethics statement

All experimental protocols were approved by the Animal Care and Use Committee in the Chinese Research Academy of Environmental Sciences. The committee specifically reviewed and approved the mortality aspects of the experimental protocol. All of the living individuals were anesthetized with 100 mg/L Tricaine for 12 min and then stored at -20°C along with all the dead individuals when the toxicity tests were finished. However, some died animals during the test were disposed without euthanasia in order to obtain accurate results according to the guidelines of the toxicity test [34, 35]. We monitored the death of the test organisms daily. No pain relievers were used during the toxicity test because they can affect the results of the test. In every test, about 70 individuals were died. All the animals used in the test are not endangered or protected species in China. The scientific activities in this study were in accordance with the Law of the People’s Republic of China on the Protection of Wildlife. In addition, the tadpoles (*Rana limnocharis*), one of the test organisms, were obtained from Beijing Olympic Park with the permission of the administration office of the park.

The described sampling work in the field was approved by the Chinese Research Academy of Environmental Sciences and the Bureau of Taihu Basin Water Resources Protection. In addition, the field sampling study did not involve endangered or protected species, and no lethal sampling was conducted.

### Collection of published toxicity data for Cu

Toxicity data to native Chinese aquatic organisms for Cu were collected from China Knowledge Resource Integrated Database (http://www.cnki.net/), the public database (http://cfpub.epa.gov/ecotox), the government documents [25] and the published literatures. Only the data with described water quality parameters including major cations, anions and dissolved organic carbon (DOC) were screened in the application of BLM to derive the criteria of CMC and CCC for Cu.

### Test chemicals and organisms

Copper sulfate pentahydrate (CuSO_4_∙5H_2_O) with analytical grade (99.0% purity) was purchased from Sinopharm Chemical Regeant Co., Ltd. (China). The reconstituted water for all toxicity tests was prepared by adding different volumes of stock solutions of CaCl_2_, MgCl, NaCl, and KCl with the reagents of analytical grade and the deionised water (the conductivity with 1.15 μS/cm) according to the reference [8]. The test water was used as the dilution water to prepare different concentrations of copper by adding CuSO_4_. The pH of reconstituted water was adjusted with HCl and NaOH to 7.8±0.2 and the water aerated for at least 24-h before test (i.e., dissolved oxygen (DO) above 90% air saturation value). The water quality parameters such as major cations (Ca^2+^, Mg^2+^, Na^+^ and K^+^), anions (SO_4_^2-^ and Cl^-^), pH, hardness and alkalinity were determined before toxicity tests.

The toxicity data on aquatic organisms from at least eight families in three different phyla are required to derive WQC of CMC [31]. In the present study, seven resident aquatic species in China were chosen to carry out acute and chronic toxicity tests in addition to the published toxicity data of Cu. The seven test aquatic organisms were the common carp (*Cyprinus carpio*), the topmouth gudgeon (*Pseudorasbora parva*), the golden fish (*Carassius auratus*), the mud fish (*Misgurnus anguillicaudatus*), the oriental river prawn (*Macrobrachium nipponense*), the tadpole (*Rana limnocharis*) and the chironomidae larvae (*Chironomus plumosus*). Prior to the toxicity tests, all the organisms were acclimatized under the test conditions for at least seven days. The organisms whose mortalities did not exceed five percent during the acclimatization could be used in the toxicity tests. All toxicity tests were carried out according to ASTM guidelines [34] and OECD guidelines[35].

### Test conditions

The toxicity tests were allocated in three replicates for each concentration and one blank control. Each test containers contained seven or ten organisms. The detailed test conditions were referred to the study by Yan et al. [36]. All tests were performed with a photoperiod of 12h light. In acute toxicity tests, none feeding for test organisms were occurred during the test periods. Test beakers were placed in the water bath at 20±2°C. Measurement of pH, dissolved oxygen and temperature were carried out daily at regular time during acute toxicity tests and at renewal of the solution during chronic tests. The abnormalities and the survival of fish were inspected at least once daily during the test at regular time.

### Acute toxicity tests

The acute toxicity tests were conducted for 96 h without renewing test solutions. The test organisms including *C*. *carpio*, *P*. *parva*, *C*. *auratus*, *M*. *anguillicaudatus* and *C*. *plumosus* were purchased from the Beijing Chaoyang Spring Flower Market. The average weight of *C*. *carpio* was 0.65 g and the average body length was 4.49±0.17 cm. The average weight of *P*. *parva* was 0.50 g and the average body length was 3.65±0.19 cm. The average weight of *C*. *auratus* was 0.60 g and the average body length was 3.89±0.15 cm. The average weight of *M*. *anguillicaudatus* was 0.50 g and the average body length was 6.10±0.23 cm. The age of *C*. *plumosus* was from two instars to three instars. Tadpoles (*R*. *limnocharis*) were obtained from the Beijing Olympic Park and had an average body weight of 0.08 g and the age in the range from one month to two months. The shrimps were purchased from the Beijing Dahongmen Jingshen Seafood Market. The age of the shrimp (*M*. *nipponense*) was in the range of one to two month, and the average weight was 0.09 g.

Seven animals were assigned in the 5 L beakers covered with watch glasses with four liters of test solutions for *P*. *parva* and *M*. *anguillicaudatus* and five liters of the solutions for *C*. *carpio* and *C*. *auratus* in the fish acute toxicity test. Seven tendipes (*C*. *plumosus*) were set in the one liter beakers with 300 ml of test solutions. Ten tadpoles (*R*. *limnocharis*) and ten shrimps (*M*. *nipponense*) were set in 2 L beakers with one liter of test solutions. Nominal exposure concentrations of Cu in the toxicity test were listed in Table 1 for all test organisms.

**Table 1:** The acute toxicity and chronic toxicity to seven native aquatic organisms and the water parameters in the tests.

No.	Species	Concentrations in the acute toxicity test (mg/L)	Concentrations in the chronic toxicity test (μg/L)	Hardness (mg/L CaCO_3_)		Temp(°C)	pH	Ca (mg/L)	Mg (mg/L)	Na (mg/L)	K (mg/L)	SO_4_^2–^ (mg/L)	Cl^−^(mg/L)	Alkalinity (mg/L CaCO_3_)	96h LC_50_ (mg/L)	28d LOEC(μg/L)	28d NOEC(μg/L)	EC_20_ (μg/L)
1	*Macrobrachium nipponense*	0, 0.02, 0.04, 0.08, 0.16, 0.32, 0.64	—		253	18.6	7.78	80.1818	12.1649	17.7074	3.0158	48.0690	73.6497	44	0.417(0.333–0.499)	—	—	—
2	*Cyprinus carpio*	0, 0.2, 0.4, 0.6, 1.0, 1.2, 1.6	—		248	18.5	7.84	80.1818	12.1649	17.7074	3.0158	48.0690	73.6497	46	0.912(0.785–1.062)	—	—	—
3	*Pseudorasbora parva*	0, 0.2, 0.4, 0.6, 1.0, 1.2, 1.6	—		238	18.4	7.73	80.1818	12.1649	17.7074	3.0158	48.0690	73.6497	47	0.347(0.287–0.406)	—	—	—
4	*Carassius auratus*	0, 0.2, 0.4, 0.8, 1.6, 3.2, 6.4	0, 7, 14, 70, 140, 700		248	18.5	7.85	80.1818	12.1649	17.7074	3.0158	48.0690	73.6497	49	1.405(1.169–1.703)	70	14	67.308
5	*Misgurnus anguillicaudatus*	0, 0.02, 0.04, 0.08, 0.16, 0.32,0.64	0, 0.12, 0.24, 1.2, 2.4, 12.0		256	19.1	7.81	80.1818	12.1649	17.7074	3.0158	48.0690	73.6497	45	0.024(0.018–0.030)	2.4	1.2	2.510
6	*Rana limnocharis*	0, 1.2, 1.8, 2.4, 3.0, 3.6, 4.2	—		251	18.4	7.74	80.1818	12.1649	17.7074	3.0158	48.0690	73.6497	42	1.504(1.354–1.639)	—	—	—
7	*Chironomus plumosus*	0, 1, 10, 100, 500, 1000,10000	—		251	18.5	7.74	80.1818	12.1649	17.7074	3.0158	48.0690	73.6497	42	>10000	—	—	—

### Chronic toxicity tests

The chronic toxicity tests were conducted for 28 days and test solutions were renewed every four days. The concentrations of Cu were 0 (control), 0.007, 0.014, 0.07, 0.14 and 0.7 μg/L for *C*. *auratus* and 0(control), 0.12, 0.24, 1.20, 2.40 and 12.0 μg/L for *M*. *anguillicaudatus* from Table 1.

### Chemical measurements

The concentrations of Cu and major cations (Ca^2+^, Mg^2+^, Na^+^ and K^+^) were analyzed by inductively coupled plasma-mass (ICP-MS, Agilent 7500a, USA). The anions (SO_4_^2-^ and Cl^-^) were analyzed by ion chromatography (Thermo ICS-2100, USA). The DOC in the test water was analyzed by the total organic carbon analyzer (O.I. Aurora 1030C, USA) using the combustion method combined with infrared detection after the samples filtered through 0.45 μm membrane. The hardness of the water samples were determined by the hardness digital titration (HACH 16900, USA) and the alkalinity were determined by the portable test instrument (HACH HQ40d, USA).

### Determination of water quality parameters in the taihu lake

**Fig 1:**
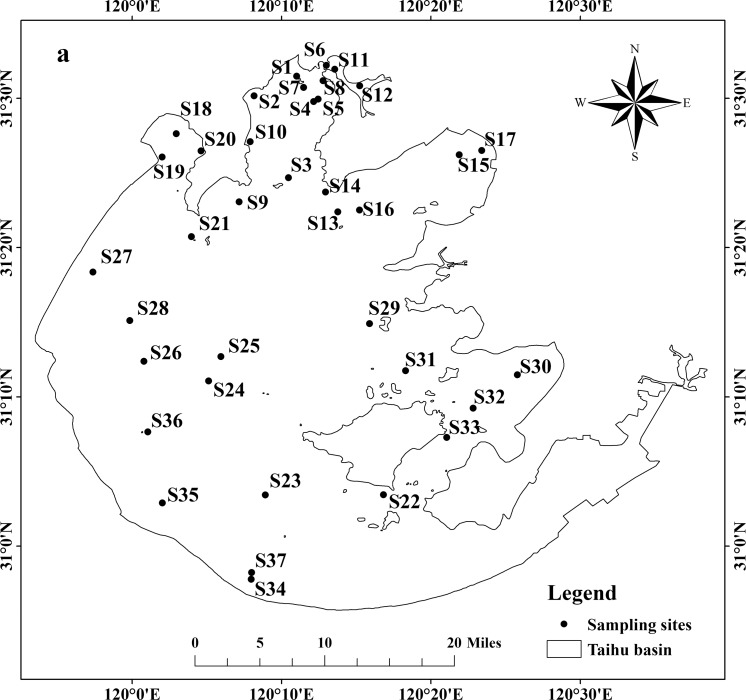
Sampling sites in Taihu Lake of China.

The water samples were collected at the depth of 50 centimeters under the water surface from 37 sampling sites (Fig 1) in the Taihu Lake (located in eastern China) in February 2014. The locations of 37 sampling sites were recorded in situ with a global positioning system (GPS). The samples were stored in 250 ml polyethylene bottles and carried back to the laboratory. The temperature and pH were determined by on-line water quality parameter instrument (HACH Hydrolab DS5, USA). The DOC in the sample determined were acidized with sulfuric acid and filtered through 0.45 μm polyethersulfone membrane. The concentrations of Cu and the water quality parameters were analyzed according to the methods described in the section 2.6. The concentration of S^2-^ below the detection limit input the default value (1×10^−10^ mg/L) of S^2-^ in the BLM.

### Data analysis and statistics

Toxicity data of Cu to native species were analyzed with probit analyses using SPSS 18.0 software for Windows. The values of 96 h LC_50_ and 95% confidence intervals and 28 d EC_20_ in the chronic toxicity test were determined. The no observed effect concentration (NOEC) and the lowest observed effect concentration (LOEC) for the most sensitive biological endpoint in each test were estimated with one-way ANOVA analyses with SPSS 18.0 software. Because the NOEC and LOEC values might vary with the the concentrations in tests [37], the EC_20_ values in the chronic tests were recommended to derive the criteria for Cu [25].

A conversion factor of 0.96 recommended by US EPA [25] was used to convert the total concentration of Cu to its dissolved concentration which could be used to develop the BLM-based criterion (CMC) for Cu. The biotic ligand model (BLM version 2.2.3) was obtained from the website (http://www.hydroqual.com/wr_blm.html). The US EPA guideline for aquatic life criteria was used to derive the WQCs of CMC and CCC for Cu in China and in the Taihu Lake [31].

## Results

### Toxicity of Cu to seven native aquatic organisms and the data collected from published literatures

The acute and chronic toxicity data for seven native aquatic organisms were shown in Table 1, and the water quality parameters in the toxicity tests were shown as well. The data collected from published literatures were shown as the supplementary information of the S1 Table. In the acute toxicity to native organisms, the most sensitive species to Cu was *M*. *anguillicaudatus* with 96h-LC_50_ of 0.024 mg/L, and the least sensitive species was *C*. *plumosus* with 96h-LC_50_ above 10000 mg/L. The toxicity of *M*. *nipponense*, *C*. *carpio* and *P*. *parva* were less sensitive and their 96h-LC_50_ values were at the same order of magnitude. In the chronic toxicity, *M*. *anguillicaudatus* was much more sensitive than *C*. *auratus*, and the difference in their 28d-NOEC values were about 12 folds and the EC_20_ values about 27 folds. Among all the acute toxicity data, the toxicity of Cu to *D*. *magna* was the highest followed by *C*. *dubia* (Table 2).

**Table 2:** The final acute values and criteria calculations using the water quality parameters (national (left) and Taihu Lake(right)).

R	Species	SMAV (μg/L)	GMAV (μg/L)	SACRs	R	Species	SMAV(μg/L)	GMAV (μg/L)	SACRs
15	*Chironomus plumosus*	> 846845.855	> 846845.855		13	*Chironomus plumosus*	> 489624.771	> 489624.771	
14	*Lepomis macrochirus*	2231.410	2231.410		12	*Rana limnocharis*	4896.248	4896.248	
13	*Rana limnocharis*	1112.011	1112.011		11	*Carassius auratus*	3971.316	3971.316	20.87
12	*Carassius auratus*	846.846	846.846	20.87	10	*Cryprinus carpiod*	2815.101	2815.101	
11	*Cryprinus carpiod*	594.896	594.896		9	*Oryzias latipes*	1687.535	1687.535	
10	*Oryzias latipes*	310.449	310.449		8	*Macrobrachium nipponense*	1623.984	1623.984	
9	*Macrobrachium nipponense*	310.106	310.106		7	*Pseudorasbora parva*	1401.922	1401.922	
8	*Pseudorasbora parva*	259.195	259.195		6	*Ctenopharyngodon idellus*	1292.327	1292.327	
7	*Ctenopharyngodon idellus*	223.852	223.852		5	*Hypophthalmichtys molitrix*	646.369	646.369	
6	*Hypophthalmichtys molitrix*	86.414	86.414		4	*Lumbriculus variegatus*	450.051	450.051	
5	*Lumbriculus variegatus*	48.413	48.413		3	*Misgurnus anguillicaudatus*	366.534	366.534	9.56
4	*Misgurnus anguillicaudatus*	34.646	34.646	9.56	2	*Ceriodaphnia dubia*	115.109	115.109	2.85
3	*Oncorhynchus gorbuscha*	40.130	31.848	2.88	1	*Daphnia magna*	111.980	111.980	3.42
	*Oncorhynchus kisutch*	22.930							
	*Oncorhynchus mykiss*	35.104							
2	*Ceriodaphnia dubia*	5.934	5.934	2.85					
1	*Daphnia magna*	5.927	5.927	3.42					
FACR = 5.62 FAV = 2.782 μg/L CMC = 1.391 μg/L CCC = FAV/FACR = 0.495 μg/L					FACR = 6.64 FAV = 64.391μg/L CMC = 32.195 μg/L CCC = FAV/FACR = 9.697μg/L				

### The national criteria for Cu based on BLM

Several water quality parameters like DOC, HA and S^2-^ in the toxicity tests with native aquatic organisms were set on their default values which were 0.05 mg/L, 10% and 1×10^−10^ mg/L, respectively. The reference exposure conditions of moderately-hard reconstituted water used to normalize the LC50s by the BLM (version 2.2.3) were as follows: temperature = 20°C, pH = 7.5, DOC = 0.5 mg/L, Ca^2+^ = 14.0 mg/L, Mg^2+^ = 12.1 mg/L, Na^+^ = 26.3 mg/L, K^+^ = 2.1 mg/L, SO_4_^2-^ = 81.4 mg/L, Cl^-^ = 1.90 mg/L, alkalinity = 65.0 mg/L and S^2-^ = 0.0003 mg/L [25]. The species mean acute values (SMAVs) were calculated as geometric means of the acute values, and the genus mean acute values (GMAVs) were calculated as geometric means of the SMAVs. The species acute-chronic ratios (SACRs) were calculated as a ratio between acute and chronic values. Ranked SMAVs and GMAVs with SACRs and final acute-chronic ratio (FACR) were listed in Table 2.

17 data including nine species of fish, five species of invertebrates, and one amphibian species were used to derive normalized LC_50_ values with the above-mentioned water quality parameters. The values of SMAVs ranged from 5.927 μg/L for the most sensitive species, *D*. *magnia*, to 846845.855 μg/L for the least sensitive species, *C*. *plumosus*. The values of 15 GMAV were obtained and ranked in Table 2 to derive the WQCs in China. The BLM-based final acute value (FAV) of Cu was 2.782 μg/L, and a CMC of 1.391 μg/L Cu was calculated by dividing FAV by two. The FACR of 5.62 was calculated as the geometric mean of the SACRs with five species, 2.85 of *C*. *dubia*, 2.88 of *O*. *gorbuscha*, 3.42 of *D*. *magna*, 9.56 of *M*. *anguillicaudatus*, and 20.87 of *C*. *auratus*. The CCC value of 0.495 μg/L was obtained by dividing the FAV (2.782 μg/L) by the FACR (3.55).

### The criteria for Cu in the taihu lake based on BLM

**Fig 2:**
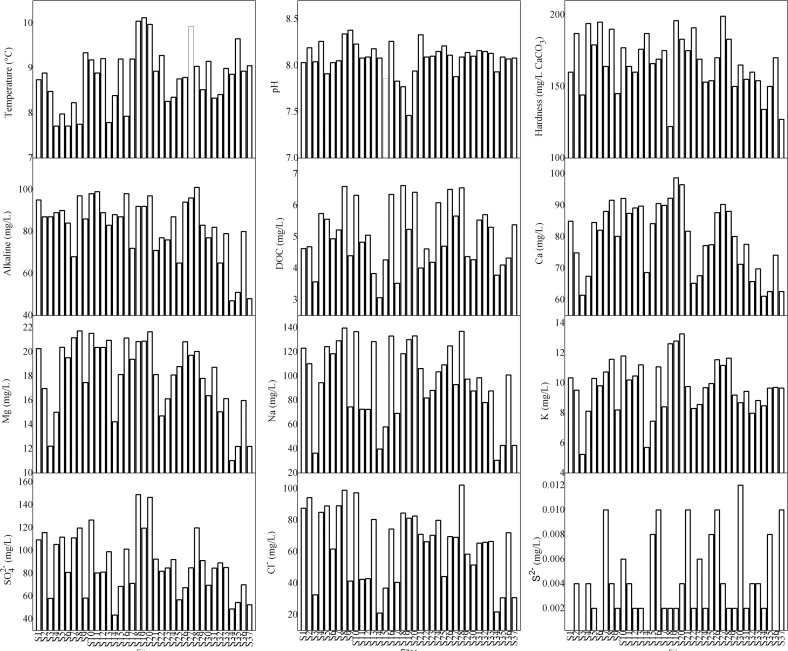
The water quality parameters and exposure concentration of copper in Taihu Lake.

The water quality parameters of samples from 37 sampling sites in the Taihu Lake were shown in Fig 2. The median values of the water quality parameters in the Taihu Lake were as follows: temperature = 8.89°C, pH = 8.09, hardness = 169 mg/L (CaCO_3_), DOC = 4.935 mg/L, Ca^2+^ = 81.75 mg/L, Mg^2+^ = 18.73 mg/L, Na^+^ = 98.70 mg/L, K^+^ = 9.70 mg/L, SO_4_^2-^ = 84.91 mg/L, Cl^-^ = 69.19 mg/L, alkalinity = 87.0 mg/L and S^2-^ = 0.004 mg/L. Using the median values of these water quality parameters in the Taihu Lake, the SMAV and GMAV values were calculated (Table 2).

13 data including seven species of fish, five species of invertebrates, and one amphibian species were used to derive normalized LC_50_ values with normalized LC_50_ values with the median water quality parameters in the Taihu Lake. The values of SMAVs ranged from 111.98 μg/L for the most sensitive species, *D*. *magnia*, to 489624.771 μg/L for the least sensitive species, *C*. *plumosus*. The values of 13 GMAV were obtained and ranked in Table 2.

The mean acute values of thirteen genera to freshwater aquatic animals were derived the WQCs in the Taihu Lake. The BLM-derived FAV of Cu was 64.391 μg/L and the CMC of Cu was 32.195 μg/L (FAV/2). The FACR of 6.64 was calculated as geometric mean of the SACRs of four species, 2.85 of *C*. *dubia*, 3.42 of *D*. *magna*, 9.56 of *M*. *anguillicaudatus*, and 20.87 of *C*. *auratus*. The CCC of 9.697 μg/L was obtained with dividing the FAV (64.391 μg/L) by the FACR (6.64).

## Discussion

In this study, Chinese native aquatic organisms were selected to supplement the toxicity data of Cu to derive the CMC and CCC by the BLM approach with the screened toxicity data from the literatures. Although in the studies a great many of toxicity data of Cu using Chinese resident species, for example, *C*. *carpio* [38], *C*. *auratus* [39], *M*. *anguillicaudatus* [40], *M*. *nipponense* [41], *R*. *limnocharis* [42] and *C*. *idellus* [43] are available, these toxicity data suitable for deriving the criteria in the freshwater for Cu by BLM are not numerous. Few of these tests were accompanied by analysis of the exposure concentrations for Cu and water quality parameters in the test water. In addition, it is difficult to directly compare toxicity data without description of water characteristics with the BLM-based toxicity data.

In accordance with the US EPA guidelines of deriving the WQCs, eight freshwater animal species on three phyla and eight families were required in waters. In the present study, the toxicity data collected in the literatures and the data in the tests could satisfy the demand. The data of the introduced species (*Lepomis macrochirus*, *Oncorhynchus gorbuscha*, *Oncorhynchus kisutch* and *Oncorhynchus mykiss*) were included to derive the national criteria because their high economic values in Chinese aquaculture. Seven native species of *Cyprinus carpio*, *Pseudorasbora parva*, *Carassius auratus*, *Misgurnus anguillicaudatus*, *Macrobrachium nipponense*, *Rana limnocharis*, and *Chironomus plumosus* were complemented to obtain the toxicity data for deriving the WQC because these species are common and widespread in China [44, 45]. The ACRs for the species of *C*. *auratus*, *M*. *anguillicaudatus*, *O*. *gorbuscha*, *C*. *dubia* and *D*. *magna* were calculated the FACR for the national criteria. The toxicity data of the introduced species (*O*. *gorbuscha*) were excluded when deriving the criteria in the Taihu Lake. The FACRs of the nation(5.62) and the Taihu Lake (6.64) were at the same order of magnitude with the values of 3.22 in the freshwater criteria of Cu by US EPA[25].

The BLM-based national criteria for Cu in the freshwater in China were obtained with the values of CMC of 1.391 μg/L and CCC of 0.495 μg/L, which were lower than those of USEPA with BLM (the CMC of 2.337 μg/L and CCC of 1.451 μg/L) [25]. The differences of the criteria for Cu in these studies were mainly attributed to differences in species selection. With the same water quality parameters, the final four GMAV values in China, which were from the normalized values of six species of (*D*. *magna*, *C*. *dubia*, *O*. *gorbuscha*, *O*. *kisutch* and *O*. *mykiss*, and *M*. *anguillicaudatus*) were higher than the final GMAV in U. S. from the normalized values of the most sensitive five species (*D*. *pulicaria* and *D*. *magna*, *C*. *dubia*, *L*. *virens* and *G*. *pseusolimnaeus*).

The site-specific criteria of Cu in the Taihu Lake of the CMC of 32.195 μg/L and CCC of 9.697 μg/L with water quality conditions of 37 sample sites were higher than the national Cu criteria. The concentrations or levels for most of water quality parameters in the Taihu Lake were higher than the values in the reference exposure conditions recommended by US EPA except for HA with the default value of 10%. Among these water quality parameters, the median concentration of DOC (4.935 mg/L) was about ten times higher than the value of 0.5 mg/L proposed by US EPA. Dissolved organic matter in water played a critical role in determining the speciation and toxicity of metal, which mitigated the metal toxicity with complexation [11, 17, 46]. The DOC significantly decreased the toxicity of Cu to the mussel (*Villosa iris*) and the cladoceran (*Ceriodaphnia dubia*), which markedly increased the acute EC50s and the chronic EC20s as the DOC increasing [47]. The comparison of species sensitivity distributions for native and non-native species for the pollutants such as pentachlorophenol[48], phenanthrene[49] and other eight priority pollutants in China[50] had been made. It had been demonstrated that there was no significant difference from native and non-native taxa and the foreign WQCs (for example, the U.S.) could provide the protection for the aquatic organisms in China. However, the freshwater WQCs for Cu in U.S. apparently would offer over-protection for the organisms in the Taihu Lake because of the difference of the water chemistry. It was necessary to develop the WQCs for Cu in the freshwater in China with site-specific water quality parameters.

In addition, there is no guidance considering the variability of the water quality parameters over time when calculating the criteria at present. The BLM-based criterion for one specific site was appropriate or not might be further discussed because the water quality parameters input in BLM were time-variable when deriving a site-specific criterion.

The criteria of Cu in the freshwater mainly have two forms, the hardness-dependent values and the BLM-based criteria, applied in the criterion recommendations. The freshwater high reliability trigger value of 1.4 μg/L is adopted by adjusted the hardness to 30 mg/L as CaCO_3_ with the chronic data relating to pH and hardness in Australia[51]. The hardness-adjusted criteria of 2~4 μg/L are employed with three hardness levels of <120, <180, >180 mg CaCO_3_/L by the assessment factor method in Canada [52]. However, the hardness-adjusted criterion values and the BLM-based values were demonstrated to be similar just with the low DOC concentration in the water, and both values in high DOC of water were had difference[25]. The hardness-adjusted criteria would provide the over- or under-protective of the aquatic organisms due to not take the co-variation of pH and DOC into account especially in natural waters. The BLM-derived criteria for Cu do not include the plant toxicity data[53, 54]. In the toxicity tests using plants, the constituents of the algal culture media could make effect on the water quality parameters (e.g., DOC, hardness and pH) input in the BLM [25]. Besides, similarly to the toxicity data of native species, the actual Cu concentrations and the water quality parameters exposed in the toxicity tests and in the dilution water were seldom detected in the tests. Wang et al. [55] analyzed the taxon sensitivity differences of the freshwater species for Cu, and the results showed that algae was the most sensitive taxon to Cu with 10% hazards concentrations (HC_10_) of 6.1 μg/L (95% confidence interval of 2.8~13 μg/L) without considering the effect of water quality parameters on the toxicity of Cu. Thus, most algae groups could be adequately protected by the CMC (1.391 μg/L) and CCC (0.495 μg/L) in China.

The CMC and CCC values of Cu in this study will provide useful data to derive national and site-specific WQCs for Cu. On the other hand, more endemic aquatic species should be tested in the development of a site-specific WQC, especially when the endemic biological composition is likely to be significantly different from regions where the WQC have already been developed.

## Conclusions

The effects of various water quality parameters on bioavailability and toxicity of Cu to aquatic organisms could be accurately predicted by the BLM approach. The acute and toxicity toxicity data of seven native Chinese species with water quality parameters were obtained in this study. The BLM-based WQCs for Cu in the freshwater were derived in China and in the Taihu Lake. The national CMC value for Cu of 1.391 μg/L and the CCC of 0.495 μg/L were obtained, and respective for 32.194 μg/L and 9.697 μg/L with the median water quality parameters at the specific sites in the Taihu Lake.

## Supporting Information

S1 Table S1 Table The toxicity data collected from the open literatures of copper input BLM. The cited references were listed at the below of the S1 Table.
(DOCX)
