# Supplementary material for: Water Quality Criteria for Copper Based on the BLM Approach in the Freshwater in China
Source: PLoS One. 2017 Feb 6;12(2):e0170105. doi: 10.1371/journal.pone.0170105 (PMC5293193; doi:10.1371/journal.pone.0170105)
Supplement: S1 Table — The cited references were listed at the below of the S1 Table. (DOCX) [file pone.0170105.s001.docx]

**Supporting Information**

**S1 Table.**

**S1 Table** **The toxicity data collected from the open literatures of copper input BLM**

| Species | Total LC_50_(μg/L) | Dissolved LC_50_(μg/L) | Hardness  (mg CaCO_3_/L) | Temp (°C) | pH | DOC  (mg/L) | Humic  Acid (%)^a^ | Ca  (mg/L) | Mg  (mg/L) | Na  (mg/L) | K  (mg/L) | SO_4_^2-^  (mg/L) | Cl^-^  (mg/L) | Alkalinty  (mg/L) | S^2-^  (mg/L)^b^ | References |
| --- | --- | --- | --- | --- | --- | --- | --- | --- | --- | --- | --- | --- | --- | --- | --- | --- |
| *Lumbriculus variegatus* | 130 | — | 290 | 25 | 6.57 | 0.5 | 10 | 47.8602 | 41.47 | 89.821 | 7.178 | 278.4 | 6.5081 | 235 | 0.0003 | [1] |
| *Lumbriculus variegatus* | 270 | — | 290 | 25 | 7.29 | 0.5 | 10 | 47.8602 | 41.47 | 89.821 | 7.178 | 278.4 | 6.5081 | 235 | 0.0003 | [1] |
| *Lumbriculus variegatus* | 500 | — | 290 | 25 | 5.25 | 0.5 | 10 | 47.8602 | 41.47 | 89.821 | 7.178 | 278.4 | 6.5081 | 235 | 0.0003 | [1] |
| *Ceriodaphnia dubia* | 19 | — | 52 | 24.5 | 7.5 | 1.1 | 10 | 15.2833 | 3.371316 | 1.5 | 0.57 | 3.8 | 1.4 | 55 | 0.0003 | [2] |
| *Ceriodaphnia dubia* | 17 | — | 52 | 24.5 | 7.5 | 1.1 | 10 | 15.2833 | 3.371316 | 1.5 | 0.57 | 3.8 | 1.4 | 55 | 0.0003 | [2] |
| *Ceriodaphnia dubia* | — | 25 | 45 | 25 | 7.72 | 1.5 | 10 | 11.0991 | 4.2075 | 9.5 | 1.6 | 46 | 34 | 39.7 | 0.0003 | [3] |
| *Ceriodaphnia dubia* | — | 17 | 45 | 25 | 7.72 | 1.5 | 10 | 11.0991 | 4.2075 | 9.5 | 1.6 | 46 | 34 | 39.7 | 0.0003 | [3] |
| *Ceriodaphnia dubia* | — | 30 | 45 | 25 | 7.72 | 1.5 | 10 | 11.0991 | 4.2075 | 9.5 | 1.6 | 46 | 34 | 39.7 | 0.0003 | [3] |
| *Ceriodaphnia dubia* | — | 24 | 45 | 25 | 7.72 | 1.5 | 10 | 11.0991 | 4.2075 | 9.5 | 1.6 | 46 | 34 | 39.7 | 0.0003 | [3] |
| *Ceriodaphnia dubia* | — | 28 | 45 | 25 | 7.72 | 1.5 | 10 | 11.0991 | 4.2075 | 9.5 | 1.6 | 46 | 34 | 39.7 | 0.0003 | [3] |
| *Ceriodaphnia dubia* | — | 32 | 45 | 25 | 7.72 | 1.5 | 10 | 11.0991 | 4.2075 | 9.5 | 1.6 | 46 | 34 | 39.7 | 0.0003 | [3] |
| *Ceriodaphnia dubia* | — | 23 | 45 | 25 | 7.72 | 1.5 | 10 | 11.0991 | 4.2075 | 9.5 | 1.6 | 46 | 34 | 39.7 | 0.0003 | [3] |
| *Ceriodaphnia dubia* | — | 20 | 45 | 25 | 7.72 | 1.5 | 10 | 11.0991 | 4.2075 | 9.5 | 1.6 | 46 | 34 | 39.7 | 0.0003 | [3] |
| *Ceriodaphnia dubia* | — | 19 | 45 | 25 | 7.72 | 1.5 | 10 | 11.0991 | 4.2075 | 9.5 | 1.6 | 46 | 34 | 39.7 | 0.0003 | [3] |
| *Ceriodaphnia dubia* | — | 26 | 94.1 | 25 | 8.15 | 2 | 10 | 23.2094 | 8.79835 | 5.2449 | 1.6 | 20.054 | 6.1705 | 69.6 | 0.0003 | [3] |
| *Ceriodaphnia dubia* | — | 21 | 94.1 | 25 | 8.15 | 2 | 10 | 23.2094 | 8.79835 | 5.2449 | 1.6 | 20.054 | 6.1705 | 69.6 | 0.0003 | [3] |
| *Ceriodaphnia dubia* | — | 27 | 94.1 | 25 | 8.15 | 2 | 10 | 23.2094 | 8.79835 | 5.2449 | 1.6 | 20.054 | 6.1705 | 69.6 | 0.0003 | [3] |
| *Ceriodaphnia dubia* | — | 37 | 94.1 | 25 | 8.15 | 2 | 10 | 23.2094 | 8.79835 | 5.2449 | 1.6 | 20.054 | 6.1705 | 69.6 | 0.0003 | [3] |
| *Ceriodaphnia dubia* | — | 34 | 94.1 | 25 | 8.15 | 2 | 10 | 23.2094 | 8.79835 | 5.2449 | 1.6 | 20.054 | 6.1705 | 69.6 | 0.0003 | [3] |
| *Ceriodaphnia dubia* | — | 67 | 179 | 25 | 8.31 | 2.3 | 10 | 50.1069 | 13.12323 | 14.32 | 2.4 | 22.673 | 10.979 | 140.1 | 0.0003 | [3] |
| *Ceriodaphnia dubia* | — | 38 | 179 | 25 | 8.31 | 2.3 | 10 | 50.1069 | 13.12323 | 14.32 | 2.4 | 22.673 | 10.979 | 140.1 | 0.0003 | [3] |
| *Ceriodaphnia dubia* | — | 78 | 179 | 25 | 8.31 | 2.3 | 10 | 50.1069 | 13.12323 | 14.32 | 2.4 | 22.673 | 10.979 | 140.1 | 0.0003 | [3] |
| *Ceriodaphnia dubia* | — | 81 | 179 | 25 | 8.31 | 2.3 | 10 | 50.1069 | 13.12323 | 14.32 | 2.4 | 22.673 | 10.979 | 140.1 | 0.0003 | [3] |
| *Ceriodaphnia dubia* | — | 28 | 97.6 | 25 | 8 | 2 | 10 | 24.0727 | 9.1256 | 5.44 | 1.6 | 20.8 | 6.4 | 74.2 | 0.0003 | [4] |
| *Ceriodaphnia dubia* | — | 84 | 182 | 25 | 8 | 2.3 | 10 | 50.9467 | 13.34317 | 14.56 | 2.4 | 23.053 | 11.163 | 144.3 | 0.0003 | [4] |
| *Ceriodaphnia dubia* | 13.4 | — | 57.1 | 25 | 8.18 | 0.5 | 10 | 9.42352 | 8.1653 | 17.685 | 1.4133 | 54.815 | 1.2814 | 81 | 0.0003 | [5] |
| *Ceriodaphnia dubia* | 6.98 | — | 80 | 20 | 7.6 | 0.5 | 10 | 13.2028 | 11.44 | 24.778 | 1.9801 | 76.799 | 1.7953 | 53 | 0.0003 | [6] |

**S1 Table** **(continued)**

| *Daphnia magna* | 9.1 | — | 39 | 20 | 7.8 | 1.1 | 10 | 10.9867 | 2.7776 | 5.8136 | 0.7 | 7.9394 | 7.7684 | 51 | 0.0003 | [7] |
| --- | --- | --- | --- | --- | --- | --- | --- | --- | --- | --- | --- | --- | --- | --- | --- | --- |
| *Daphnia magna* | 11.7 | — | 39 | 20 | 7.8 | 1.1 | 10 | 10.9867 | 2.7776 | 5.8136 | 0.7 | 7.9394 | 7.7684 | 51 | 0.0003 | [7] |
| *Daphnia magna* | 6.6 | — | 39 | 20 | 7.79 | 1.1 | 10 | 10.7129 | 2.7203 | 5.7423 | 0.7 | 7.6578 | 7.6406 | 50 | 0.0003 | [7] |
| *Daphnia magna* | 9.9 | — | 39 | 20 | 7.79 | 1.1 | 10 | 10.7129 | 2.7203 | 5.7423 | 0.7 | 7.6578 | 7.6406 | 50 | 0.0003 | [7] |
| *Daphnia magna* | 11.7 | — | 39 | 20 | 6.9 | 1.1 | 10 | 10.9867 | 2.7776 | 5.8136 | 0.7 | 7.9394 | 7.7684 | 30 | 0.0003 | [7] |
| *Daphnia magna* | 6.7 | — | 39 | 20 | 6.9 | 1.1 | 10 | 10.9867 | 2.7776 | 5.8136 | 0.7 | 7.9394 | 7.7684 | 30 | 0.0003 | [7] |
| *Daphnia magna* | 9.1 | — | 26 | 20 | 7.6 | 1.1 | 10 | 7.4273 | 2.0327 | 4.8867 | 0.7 | 4.2786 | 6.107 | 24 | 0.0003 | [7] |
| *Daphnia magna* | 5.2 | — | 27 | 20 | 7.7 | 1.1 | 10 | 7.7011 | 2.09 | 4.958 | 0.7 | 4.5602 | 6.2348 | 24 | 0.0003 | [7] |
| *Daphnia magna* | 41.2 | — | 170 | 20 | 7.8 | 0.5 | 10 | 27.9433 | 24.23527 | 52.507 | 4.1961 | 162.74 | 3.8045 | 115 | 0.0003 | [8] |
| *Daphnia magna* | 10.5 | — | 170 | 20 | 7.8 | 0.5 | 10 | 27.9433 | 24.23527 | 52.507 | 4.1961 | 162.74 | 3.8045 | 115 | 0.0003 | [8] |
| *Daphnia magna* | 20.6 | — | 170 | 20 | 7.8 | 0.5 | 10 | 27.9433 | 24.23527 | 52.507 | 4.1961 | 162.74 | 3.8045 | 115 | 0.0003 | [8] |
| *Daphnia magna* | 17.3 | — | 170 | 20 | 7.8 | 0.5 | 10 | 27.9433 | 24.23527 | 52.507 | 4.1961 | 162.74 | 3.8045 | 115 | 0.0003 | [8] |
| *Daphnia magna* | 70.7 | — | 170 | 20 | 7.8 | 0.5 | 10 | 27.9433 | 24.23527 | 52.507 | 4.1961 | 162.74 | 3.8045 | 115 | 0.0003 | [8] |
| *Daphnia magna* | 31.3 | — | 170 | 20 | 7.8 | 0.5 | 10 | 27.9433 | 24.23527 | 52.507 | 4.1961 | 162.74 | 3.8045 | 115 | 0.0003 | [8] |
| *Daphnia magna* | 7.1 | — | 109.9 | 21 | 6.93 | 2.4 | 10 | 40 | 2.43 | 85.1 | 1.23 | 10 | 106 | 12.5 | 0.0003 | [9] |
| *Daphnia magna* | 16.4 | — | 109.9 | 21 | 6.93 | 3.4 | 10 | 40 | 2.43 | 85.1 | 1.23 | 10 | 106 | 12.5 | 0.0003 | [9] |
| *Daphnia magna* | 39.9 | — | 109.9 | 21 | 7.43 | 3.4 | 10 | 40 | 2.43 | 85.1 | 1.23 | 10 | 106 | 13.875 | 0.0003 | [9] |
| *Daphnia magna* | 18.7 | — | 109.9 | 21 | 7.43 | 2.4 | 10 | 40 | 2.43 | 85.1 | 1.23 | 10 | 106 | 13.875 | 0.0003 | [9] |
| *Daphnia magna* | 18.9 | — | 109.9 | 21 | 7.82 | 2.4 | 10 | 40 | 2.43 | 85.1 | 1.23 | 10 | 106 | 14.5 | 0.0003 | [9] |
| *Daphnia magna* | 39.7 | — | 109.9 | 21 | 7.82 | 3.4 | 10 | 40 | 2.43 | 85.1 | 1.23 | 10 | 106 | 14.5 | 0.0003 | [9] |
| *Daphnia magna* | 46 | — | 109.9 | 21 | 6.93 | 4.4 | 10 | 40 | 2.43 | 85.1 | 1.23 | 10 | 106 | 12.5 | 0.0003 | [9] |
| *Daphnia magna* | 71.9 | — | 109.9 | 21 | 6.93 | 6.1 | 10 | 40 | 2.43 | 85.1 | 1.23 | 10 | 106 | 12.5 | 0.0003 | [9] |
| *Daphnia magna* | 57.2 | — | 109.9 | 21 | 7.43 | 4.4 | 10 | 40 | 2.43 | 85.1 | 1.23 | 10 | 106 | 13.875 | 0.0003 | [9] |
| *Daphnia magna* | 67.8 | — | 109.9 | 21 | 7.82 | 4.4 | 10 | 40 | 2.43 | 85.1 | 1.23 | 10 | 106 | 14.5 | 0.0003 | [9] |
| *Daphnia magna* | 26 | — | 52 | 18.2 | 7.8 | 1.1 | 10 | 14 | 3.5 | 12 | 2.9 | 23 | 11 | 45 | 0.0003 | [10] |
| *Daphnia magna* | 30 | — | 105 | 20.3 | 7.9 | 1.1 | 10 | 29 | 6.8 | 29 | 5.3 | 57 | 21 | 79 | 0.0003 | [10] |
| *Daphnia magna* | 38 | — | 106 | 19.7 | 8.1 | 1.1 | 10 | 29 | 6.8 | 29 | 5.3 | 57 | 21 | 82 | 0.0003 | [10] |
| *Daphnia magna* | 69 | — | 207 | 19.9 | 8.3 | 1.1 | 10 | 58 | 13 | 62 | 8.2 | 127 | 40 | 166 | 0.0003 | [10] |
| *Daphnia magna* | 4.8 | — | 7.1 | 24 | 8.55 | 0.5 | 10 | 1.15182 | 1.027387 | 3.5102 | 2.8052 | 6.8159 | 2.5434 | 56 | 0.0003 | [10] |
| *Daphnia magna* | 7.4 | — | 20.6 | 24 | 6.97 | 0.5 | 10 | 3.39973 | 2.9458 | 2.5478 | 2.1356 | 19.776 | 1.9363 | 60 | 0.0003 | [10] |
| *Daphnia magna* | 6.5 | — | 23 | 24 | 8.52 | 0.5 | 10 | 3.79581 | 3.289 | 2.8446 | 2.3845 | 22.08 | 2.1619 | 64 | 0.0003 | [10] |
| *Daphnia magna* | 197.53 | — | — | 21 | 7.57 | 11.12 | 10 | 77.13 | 1.47 | 50.47 | 0.46 | 55 | 33 | 142 | — | [11] |
| *Daphnia magna* | 134.55 | — | — | 21 | 7.98 | 4.99 | 10 | 66.35 | 179.2 | 35.31 | 0.51 | 76 | 43.8 | 193 | — | [11] |

**S1 Table** **(continued)**

| *Daphnia magna* | 244.45 | — | — | 21 | 8 | 20.12 | 10 | 64.47 | 1.66 | 126.7 | 0.47 | 52 | 57.6 | 219 | — | [11] |
| --- | --- | --- | --- | --- | --- | --- | --- | --- | --- | --- | --- | --- | --- | --- | --- | --- |
| *Daphnia magna* | 155.69 | — | — | 21 | 8.07 | 10.84 | 10 | 78.59 | 194.6 | 64.93 | 0.53 | 90 | 72.4 | 258 | — | [11] |
| *Daphnia magna* | 350 | — | — | 21 | 7.98 | 11.95 | 10 | 46.54 | 1.01 | 70.01 | 0.59 | 63 | 50.8 | 69 | — | [11] |
| *Daphnia magna* | 210.49 | — | — | 21 | 7.72 | 7.31 | 10 | 104.9 | 37.69 | 128.4 | 7.31 | 67 | 66.2 | 87 | — | [11] |
| *Daphnia magna* | 194.78 | — | — | 21 | 7.56 | 10.06 | 10 | 53.01 | 1.01 | 58.54 | 0.6 | 55 | 44 | 79 | — | [11] |
| *Daphnia magna* | 187.77 | — | — | 21 | 7.86 | 6.44 | 10 | 111.8 | 43.36 | 140.9 | 15.46 | 66 | 54 | 98 | — | [11] |
| *Daphnia magna* | 71.46 | — | — | 21 | 8.4 | 1.9 | 10 | 4 | 0.5 | 0.75 | 0.35 | 0.1 | 2 | 48 | — | [11] |
| ***Oncorhynchus gorbuscha*** | 143 | — | 83.1 | 7.15 | 7.63 | 2.58 | 10 | 22.3428 | 6.313221 | 10.259 | 7.5024 | 25.1 | 9.994 | 62.5 | 0.0003 | [10] |
| ***Oncorhynchus gorbuscha*** | 87 | — | 83.1 | 7.15 | 7.63 | 2.58 | 10 | 22.3428 | 6.313221 | 10.259 | 7.5024 | 25.1 | 9.994 | 62.5 | 0.0003 | [10] |
| ***Oncorhynchus gorbuscha*** | 199 | — | 83.1 | 7.15 | 7.63 | 2.58 | 10 | 22.3428 | 6.313221 | 10.259 | 7.5024 | 25.1 | 9.994 | 62.5 | 0.0003 | [10] |
| ***Oncorhynchus kisutch*** | 164 | — | 33 | 13.5 | 7.29 | 2.496 | 10 | 8.77741 | 2.698479 | 7.3188 | 1.15 | 6.1426 | 6.8124 | 29 | 0.0003 | [12] |
| ***Oncorhynchus kisutch*** | 33 | — | 25 | 12 | 7.3 | 1.3 | 10 | 6.8 | 1.8 | 5 | 0.6 | 4.2 | 6 | 24 | 0.0003 | [13] |
| ***Oncorhynchus kisutch*** | 46 | — | 20 | 9.4 | 7.29 | 1.3 | 10 | 5.7845 | 1.6889 | 4.4589 | 0.7 | 2.589 | 5.3402 | 22 | 0.0003 | [14] |
| ***Oncorhynchus kisutch*** | 61 | 49 | 31.1 | 13.3 | 7.3 | 3.2 | 10 | 8.01999 | 2.695987 | 5.12 | 0.653 | 4 | 4.5 | 29.6 | 0.0003 | [15] |
| ***Oncorhynchus kisutch*** | 63 | 51 | 31.1 | 13.3 | 7.3 | 3.2 | 10 | 8.01999 | 2.695987 | 5.12 | 0.653 | 4 | 4.5 | 29.6 | 0.0003 | [15] |
| ***Oncorhynchus kisutch*** | 86 | 58 | 31.6 | 15.7 | 7.5 | 3.2 | 10 | 8.14893 | 2.739331 | 5.12 | 0.653 | 3.5 | 4.2 | 30.4 | 0.0003 | [15] |
| ***Oncorhynchus kisutch*** | 103 | 78 | 31 | 15.3 | 7.2 | 3.2 | 10 | 7.99421 | 2.687318 | 5.12 | 0.653 | 2.3 | 3.1 | 29.7 | 0.0003 | [15] |
| ***Oncorhynchus mykiss*** | 110 |  | 169 | 12 | 8.2 | 0.5 | 10 | 27.891 | 24.167 | 52.344 | 4.183 | 162.24 | 3.7927 | 117 | 0.0003 | [10] |
| ***Oncorhynchus mykiss*** | 50 |  | 169 | 12 | 7.95 | 0.5 | 10 | 27.891 | 24.167 | 52.344 | 4.183 | 162.24 | 3.7927 | 117 | 0.0003 | [10] |
| ***Oncorhynchus mykiss*** | 60 |  | 169 | 12 | 7.95 | 0.5 | 10 | 27.891 | 24.167 | 52.344 | 4.183 | 162.24 | 3.7927 | 117 | 0.0003 | [10] |
| ***Oncorhynchus mykiss*** | 46.7 | 40 | 44.1 | 11.5 | 7.7 | 2 | 10 | 9.07 | 4.1 | 4.75 | 1.02 | 3.3 | 1.56 | 49.7 | 0.0003 | [10] |
| ***Oncorhynchus mykiss*** | 24.2 | 19 | 44.6 | 11.5 | 7.8 | 0.99 | 10 | 7.37 | 6.1 | 6.24 | 0.8 | 1.31 | 3.82 | 53.1 | 0.0003 | [10] |
| ***Oncorhynchus mykiss*** | — | 3.4 | 38.7 | 12 | 7.62 | 0.33 | 10 | 2.37 | 8.65 | 13.7 | 0.15 | 0.36 | 20.3 | 40 | 0.0003 | [16] |
| ***Oncorhynchus mykiss*** | — | 8.1 | 39.3 | 12 | 7.61 | 0.36 | 10 | 14.1 | 1.8 | 13.2 | 0.1 | 0.36 | 19.9 | 41.7 | 0.0003 | [16] |
| ***Oncorhynchus mykiss*** | — | 17.2 | 89.5 | 12 | 8.21 | 0.345 | 10 | 15 | 11.85 | 10.05 | 1 | 0.36 | 6.73 | 97.5 | 0.0003 | [16] |
| ***Oncorhynchus mykiss*** | — | 32 | 89.67 | 12 | 8.15 | 0.345 | 10 | 28.9 | 3.15 | 32.5 | 0.5 | 0.36 | 45.2 | 97.25 | 0.0003 | [16] |
| ***Oncorhynchus mykiss*** | 28 | — | 23 | 12.2 | 7.1 | 1.4 | 10 | 6.1 | 1.8 | 4.4 | 0.4 | 5.8 | 6 | 22 | 0.0003 | [13, 17] |
| ***Oncorhynchus mykiss*** | 17 | — | 23 | 12.2 | 7.1 | 1.4 | 10 | 6.1 | 1.8 | 4.4 | 0.4 | 5.8 | 6 | 22 | 0.0003 | [13, 17] |
| ***Oncorhynchus mykiss*** | 18 | — | 23 | 12.2 | 7.4 | 1.3 | 10 | 6.8 | 1.8 | 5 | 0.6 | 4.2 | 6 | 22 | 0.0003 | [13, 17] |
| ***Oncorhynchus mykiss*** | 29 | — | 23 | 12.2 | 7.1 | 1.3 | 10 | 6.8 | 1.8 | 5 | 0.6 | 4.2 | 6 | 22 | 0.0003 | [13, 17] |
| ***Oncorhynchus mykiss*** | — | 169 | 194 | 12.8 | 7.84 | 3.3 | 10 | 55.1 | 13.7 | 4 | 0.64 | 10 | 0.44 | 174 | 0.0003 | [18] |
| ***Oncorhynchus mykiss*** | — | 85.3 | 194 | 12.8 | 7.84 | 3.3 | 10 | 55.1 | 13.7 | 4 | 0.64 | 10 | 0.44 | 174 | 0.0003 | [18] |
| ***Oncorhynchus mykiss*** | — | 83.3 | 194 | 12.8 | 7.84 | 3.3 | 10 | 55.1 | 13.7 | 4 | 0.64 | 10 | 0.44 | 174 | 0.0003 | [18] |

**S1 Table** **(continued)**

| ***Oncorhynchus mykiss*** | — | 103 | 194 | 12.8 | 7.84 | 3.3 | 10 | 55.1 | 13.7 | 4 | 0.64 | 10 | 0.44 | 174 | 0.0003 | [18] |
| --- | --- | --- | --- | --- | --- | --- | --- | --- | --- | --- | --- | --- | --- | --- | --- | --- |
| ***Oncorhynchus mykiss*** | — | 274 | 194 | 12.8 | 7.84 | 3.3 | 10 | 55.1 | 13.7 | 4 | 0.64 | 10 | 0.44 | 174 | 0.0003 | [18] |
| ***Oncorhynchus mykiss*** | — | 128 | 194 | 12.8 | 7.84 | 3.3 | 10 | 55.1 | 13.7 | 4 | 0.64 | 10 | 0.44 | 174 | 0.0003 | [18] |
| ***Oncorhynchus mykiss*** | — | 221 | 194 | 12.8 | 7.84 | 3.3 | 10 | 55.1 | 13.7 | 4 | 0.64 | 10 | 0.44 | 174 | 0.0003 | [18] |
| ***Oncorhynchus mykiss*** | — | 165 | 194 | 12.8 | 7.84 | 3.3 | 10 | 55.1 | 13.7 | 4 | 0.64 | 10 | 0.44 | 174 | 0.0003 | [18] |
| ***Oncorhynchus mykiss*** | — | 197 | 194 | 12.8 | 7.84 | 3.3 | 10 | 55.1 | 13.7 | 4 | 0.64 | 10 | 0.44 | 174 | 0.0003 | [18] |
| ***Oncorhynchus mykiss*** | — | 514 | 194 | 12.8 | 7.84 | 3.3 | 10 | 55.1 | 13.7 | 4 | 0.64 | 10 | 0.44 | 174 | 0.0003 | [18] |
| ***Oncorhynchus mykiss*** | — | 243 | 194 | 12.8 | 7.84 | 3.3 | 10 | 55.1 | 13.7 | 4 | 0.64 | 10 | 0.44 | 174 | 0.0003 | [18] |
| ***Oncorhynchus mykiss*** | 2.8 | — | 9.2 | 15.5 | 6.96 | 0.5 | 10 | 2.3 | 0.7 | 2 | 0.2 | 4.6 | 2.1 | 11 | 0.0003 | [19] |
| ***Oncorhynchus mykiss*** | 90 | 68 | 31 | 15.3 | 7.2 | 3.2 | 10 | 7.99421 | 2.687318 | 5.12 | 0.653 | 2.3 | 3.1 | 29.7 | 0.0003 | [15] |
| ***Oncorhynchus mykiss*** | 19.6 | 18 | 36.1 | 11.4 | 7.6 | 1.31 | 10 | 4.03 | 7.13 | 1.56 | 0.26 | 1.49 | 0.88 | 36.6 | 0.0003 | [10] |
| ***Oncorhynchus mykiss*** | 12.9 | 12 | 36.2 | 11.5 | 6.1 | 1.36 | 10 | 3.93 | 7.27 | 1.57 | 0.28 | 1.47 | 0.87 | 8.5 | 0.0003 | [10] |
| ***Oncorhynchus mykiss*** | 5.9 | 5.7 | 20.4 | 11.7 | 7.5 | 0.15 | 10 | 3.13 | 2.77 | 2.62 | 0.25 | 0.36 | 1.48 | 23 | 0.0003 | [10] |
| ***Oncorhynchus mykiss*** | 37.8 | 35 | 45.2 | 11.7 | 7.7 | 1.23 | 10 | 9.7 | 4.43 | 5.33 | 0.97 | 3.41 | 1.47 | 50 | 0.0003 | [10] |
| ***Oncorhynchus mykiss*** | 25.1 | 18 | 45.4 | 11.8 | 6.3 | 1.22 | 10 | 9.7 | 4.43 | 5.02 | 0.98 | 3.37 | 1.37 | 10.9 | 0.0003 | [10] |
| ***Oncorhynchus mykiss*** | 17.2 | 17 | 41.9 | 12.3 | 7.9 | 0.33 | 10 | 6.6 | 5.97 | 5.89 | 0.63 | 1.11 | 3.37 | 48.3 | 0.0003 | [10] |
| ***Oncorhynchus mykiss*** | 101 | — | 214 | 7.64 | 7.94 | 0.27 | 10 | 49.4 | 24.1 | 10.3 | 1.75 | 18.9 | 5.28 | 198 | 0.0003 | [10] |
| ***Oncorhynchus mykiss*** | 308 | — | 220 | 7.74 | 7.92 | 0.36 | 10 | 51.2 | 25.5 | 8.36 | 2.1 | 24 | 4.64 | 197 | 0.0003 | [10] |
| ***Oncorhynchus mykiss*** | 93 | — | 105 | 7.77 | 7.82 | 0.1 | 10 | 23.1 | 11.8 | 3.54 | 3.22 | 17.1 | 2.91 | 94.1 | 0.0003 | [10] |
| ***Oncorhynchus mykiss*** | 35.9 | — | 98.2 | 8.49 | 7.89 | 0.045 | 10 | 22.3 | 11.2 | 3.58 | 0.9 | 11.5 | 2.85 | 87.9 | 0.0003 | [10] |
| ***Oncorhynchus mykiss*** | 54.4 |  | 104 | 16.3 | 7.83 | 0.28 | 10 | 22.4 | 11.4 | 3.76 | 2.72 | 12.4 | 3.01 | 97.6 | 0.0003 | [10] |
| ***Oncorhynchus mykiss*** | 260 | 249.6 | — | 15.7 | 7.5 | 2.61 | — | 22.7 | 2.49 | 0.49 | 1.02 | 13.72 | 0.84 | 165 | — | [20] |
| ***Oncorhynchus mykiss*** | 280 | 268.8 | — | 15.4 | 7.5 | 3.05 | — | 22.9 | 2.56 | 4.08 | 2.05 | 4.3 | 5.11 | 118.6 | — | [20] |
| ***Oncorhynchus mykiss*** | 270 | 259.2 | — | 15.6 | 6.79 | 2.81 | — | 17.4 | 2.38 | 8.5 | 6.99 | 45.38 | 24.97 | 133.2 | — | [20] |
| ***Oncorhynchus mykiss*** | 290 | 278.4 | — | 15.4 | 7.07 | 3.85 | — | 30.5 | 4.6 | 0.57 | 0.83 | 148.5 | 0.8 | 148.7 | — | [20] |
| ***Oncorhynchus mykiss*** | 150 | 144 | — | 15.7 | 7.46 | 5.9 | — | 28.7 | 3.47 | 12.1 | 22 | 915.7 | 59.2 | 89.9 | — | [20] |
| ***Oncorhynchus mykiss*** | 200 | 192 | — | 18.6 | 7.43 | 3.87 | — | 19 | 6.9 | 7.71 | 0.66 | 21.9 | 0.44 | 125.7 | 0.009 | [20] |
| ***Oncorhynchus mykiss*** | 220 | 211.2 | — | 15.8 | 7.5 | 3.71 | — | 19 | 5.8 | 6.2 | 0.8 | 20.6 | 0.39 | 131 | — | [20] |
| ***Oncorhynchus mykiss*** | 470 | 451.2 | — | 14.5 | 7.68 | 3.78 | — | 19 | 5.1 | 6.7 | 0.8 | 46.47 | 1.54 | 109.6 | — | [20] |
| ***Oncorhynchus mykiss*** | 280 | 268.8 | — | 17.1 | 7.13 | 2.02 | — | 47 | 1.75 | 8.2 | 2.36 | 28.8 | 17.42 | 118.2 | — | [20] |
| ***Oncorhynchus mykiss*** | 240 | 230.4 | — | 17.6 | 7.28 | 1.51 | — | 33 | 2.17 | 6.85 | 2.43 | 26.84 | 7.93 | 134.4 | — | [20] |
| ***Oncorhynchus mykiss*** | 170 | 163.2 | — | 17 | 7.41 | 2.14 | — | 25.6 | 2.43 | 6.42 | 195 | 22.35 | 8.6 | 95 | — | [20] |
| ***Oncorhynchus mykiss*** | 130 | 124.8 | — | 16.1 | 7.05 | 2.3 | — | 22.43 | 2.46 | 0.85 | 2.98 | 27.57 | 28.99 | 160 | — | [20] |

**S1 Table** **(continued)**

| ***Oncorhynchus mykiss*** | 340 | 326.4 | — | 16.5 | 7.49 | 3.57 | — | 51.03 | 2.47 | 1.23 | 4.16 | 28.24 | 27.31 | 160.1 | — | [20] |
| --- | --- | --- | --- | --- | --- | --- | --- | --- | --- | --- | --- | --- | --- | --- | --- | --- |
| ***Oncorhynchus mykiss*** | 220 | 211.2 | — | 16.4 | 7.75 | 2.23 | — | 30.29 | 1.97 | 1.78 | 5.89 | 73.7 | 69.51 | 111.2 | — | [20] |
| ***Oncorhynchus mykiss*** | 240 | 230.4 | — | 16.7 | 6.83 | 7.82 | — | 16.71 | 3.56 | 5.74 | 2.74 | 488.5 | N.D | 111.23 | — | [20] |
| ***Oncorhynchus mykiss*** | 280 | 268.8 | — | 16.9 | 7.47 | 4.72 | — | 22.1 | 3.52 | 6.49 | 4.35 | 26.08 | 7.54 | 99.64 | — | [20] |
| ***Oncorhynchus mykiss*** | 240 | 230.4 | — | 17.9 | 7.69 | 4.02 | — | 17.92 | 3.61 | 7.2 | 3.19 | N.D | 13.11 | 64.88 | — | [20] |
| ***Oncorhynchus mykiss*** | 170 | 163.2 | — | 15.8 | 7.56 | 2.31 | — | 12.77 | 3.76 | 10.32 | 1.63 | 17.7 | 11.3 | 67.13 | — | [20] |
| ***Oncorhynchus mykiss*** | 110 | 105.6 | — | 15.8 | 7.5 | 2.9 | — | 8.82 | 3.28 | 4.69 | 1.99 | 223.8 | 72.3 | 49.15 | — | [20] |
| ***Oncorhynchus mykiss*** | 200 | 192 | — | 17.8 | 6.85 | 3.5 | — | 10 | 3.2 | 9 | 0.95 | 119 | 5.93 | 77.6 | — | [20] |
| ***Oncorhynchus mykiss*** | 230 | 220.8 | — | 15.7 | 7.69 | 3.42 | — | 13.6 | 3.53 | 2.23 | 2.68 | 29.4 | 16.8 | 79.9 | — | [20] |
| ***Oncorhynchus mykiss*** | 210 | 201.6 | — | 16.9 | 8.32 | 2.3 | — | 36.7 | 1.23 | 7.8 | 1.6 | 2.86 | 1.68 | 124 | 0.002 | [20] |
| ***Oncorhynchus mykiss*** | 320 | 307.2 | — | 16.9 | 8.32 | 3.57 | — | 48.26 | 2.17 | 4.04 | 5.44 | 12.01 | 13.06 | 160.9 | 0.004 | [20] |
| ***Oncorhynchus mykiss*** | 280 | 268.8 | — | 17 | 8.07 | 2.23 | — | 102.5 | 2.63 | 4.43 | 10.59 | 13.09 | 0.96 | 179.9 | 0.002 | [20] |
| ***Oncorhynchus mykiss*** | 330 | 316.8 | — | 16.2 | 7.38 | 3.26 | — | 34.99 | 2.81 | 9 | 5.06 | 227.9 | 10.8 | 184.2 | 0.008 | [20] |
| ***Oncorhynchus mykiss*** | 290 | 278.4 | — | 14.8 | 7.24 | 4.55 | — | 37 | 18 | 57 | 1.6 | 55.6 | 4.01 | 171.5 | — | [20] |
| ***Lepomis macrochirus*** | — | 2200 | 85 | 20.2 | 7.3 | 1.1 | 10 | 23.9 | 6.5 | 0.64 | 0.46 | 4.32 | 1.5 | 82 | 0.0003 | [10] |
| ***Lepomis macrochirus*** | 1100 | — | 45 | 20 | 7.5 | 1.1 | 10 | 13.2259 | 2.917485 | 1.3 | 0.57 | 3.4 | 1.2 | 43 | 0.0003 | [10] |
| ***Lepomis macrochirus*** | 1000 | — | 25.9 | 19 | 7.03 | 1.5 | 10 | 6.38814 | 2.42165 | 5.4743 | 1.6 | 26.489 | 19.425 | 27.1 | 0.0003 | [21] |
| ***Lepomis macrochirus*** | — | 1300 | 85 | 21.85 | 7.45 | 1.1 | 10 | 23.9 | 6.5 | 0.64 | 0.46 | 4.32 | 1.5 | 82 | 0.0003 | [10] |
| *Oryzias latipes* | 351.96 | 337.8816 | — | 23 | 7.84 | 0.05 | — | 40 | 12.03 | 17.25 | 2.88 | 48.12 | 73.48 | 45.54 | 0.0003 | [22] |
| *Oryzias latipes* | 360.9 | 346.464 | — | 23 | 7.88 | 0.05 | — | 80.12 | 12.03 | 17.25 | 2.88 | 48.12 | 144.54 | 41.83 | 0.0003 | [22] |
| *Oryzias latipes* | 435.56 | 418.1376 | — | 23 | 7.57 | 0.05 | — | 80.12 | 30 | 17.25 | 2.88 | 120 | 144.54 | 40.46 | 0.0003 | [22] |
| *Oryzias latipes* | 1086 | 1042.56 | — | 23.5 | 7.74 | 3.6 | — | 42.51 | 8.41 | 15.33 | 0.015 | 35.26 | 17.24 | 103.07 | 0.0003 | [22] |
| *Oryzias latipes* | 1314 | 1261.44 | — | 21 | 7.79 | 8.9 | — | 28.91 | 6.28 | 8.71 | 0.015 | 25.23 | 15.5 | 82.45 | 0.0003 | [22] |
| *Oryzias latipes* | 1143 | 1097.28 | — | 24.2 | 8.1 | 2.9 | — | 46.4 | 15.73 | 9.04 | 7.47 | 62.84 | 37.29 | 135.59 | 0.0003 | [22] |
| *Oryzias latipes* | 1326 | 1272.96 | — | 23.8 | 8.21 | 4.3 | — | 42.95 | 19.24 | 9.42 | 8.7 | 96.07 | 63.93 | 142.73 | 0.0003 | [22] |
| *Oryzias latipes* | 296.4 | 284.544 | — | 24 | 8.03 | 0.9 | — | 19.32 | 2.28 | 3.71 | 2.09 | 10.77 | 4.84 | 85.64 | 0.0003 | [22] |
| *Oryzias latipes* | 305.9 | 293.664 | — | 24.6 | 7.85 | 0.9 | — | 20.2 | 2.69 | 6.13 | 3.16 | 20.25 | 15.01 | 64.23 | 0.0003 | [22] |
| *Oryzias latipes* | 565.3 | 542.688 | — | 22 | 7.56 | 4.2 | — | 2.56 | 0.65 | 4.47 | 2.92 | 56.73 | 18.17 | 80.15 | 0.0003 | [22] |
| *Oryzias latipes* | 517.1 | 496.416 | — | 23 | 7.41 | 4.9 | — | 2.42 | 0.65 | 4.48 | 2.98 | 59.34 | 20.78 | 78.35 | 0.0003 | [22] |
| *Oryzias latipes* | 468.9 | 450.144 | — | 27.9 | 7.47 | 4 | — | 2.39 | 0.65 | 4.48 | 2.96 | 47.52 | 23.66 | 80.34 | 0.0003 | [22] |
| *Oryzias latipes* | 455.2 | 436.992 | — | 24.2 | 7.34 | 4 | — | 2.36 | 0.65 | 4.5 | 3.05 | 51.52 | 31.76 | 80.15 | 0.0003 | [22] |
| *Oryzias latipes* | 616.5 | 591.84 | — | 20.2 | 7.35 | 4 | — | 2.35 | 0.65 | 4.5 | 3.06 | 51.84 | 30.57 | 84.5 | 0.0003 | [22] |
| *Oryzias latipes* | 819.8 | 787.008 | — | 23 | 7.45 | 2.9 | — | 2.34 | 0.65 | 4.48 | 2.91 | 61.08 | 45.24 | 86.92 | 0.0003 | [22] |

**S1 Table** **(continued)**

| *Hypophthalmichtys molitrix* | 365.7 | 351.072 | — | 21 | 8.1 | 0.05 | 10 | 44.9 | 10.73 | 69.5 | 10 | 42.92 | 88.8 | 138.58 | 0.0003 | [23] |
| --- | --- | --- | --- | --- | --- | --- | --- | --- | --- | --- | --- | --- | --- | --- | --- | --- |
| *Hypophthalmichtys molitrix* | 425.48 | 408.4608 | — | 21 | 8.3 | 1 | 10 | 44.9 | 10.73 | 69.5 | 10 | 42.92 | 88.8 | 138.58 | 0.0003 | [23] |
| *Hypophthalmichtys molitrix* | 270.04 | 259.2384 | — | 21 | 8.3 | 0.5 | 10 | 44.9 | 10.73 | 69.5 | 10 | 42.92 | 88.8 | 138.58 | 0.0003 | [23] |
| *Hypophthalmichtys molitrix* | 639.77 | 614.1792 | — | 21 | 8.3 | 2 | 10 | 44.9 | 10.73 | 69.5 | 10 | 42.92 | 88.8 | 138.58 | 0.0003 | [23] |
| *Hypophthalmichtys molitrix* | 1138.78 | 1093.2288 | — | 21 | 8.3 | 4 | 10 | 44.9 | 10.73 | 69.5 | 10 | 42.92 | 88.8 | 138.58 | 0.0003 | [23] |
| *Ctenopharyngodon idellus* | 2364.09 | 2269.5264 | — | 22 | 7.9 | 4 | 10 | 44.9 | 10.73 | 70.65 | 10 | 42.92 | 88.8 | 138.58 | 0.0003 | [23] |
| *Ctenopharyngodon idellus* | 917.89 | 881.1744 | — | 22.1 | 8 | 2 | 10 | 44.9 | 10.73 | 61.45 | 10 | 42.92 | 88.8 | 138.58 | 0.0003 | [23] |
| *Ctenopharyngodon idellus* | 846.06 | 812.2176 | — | 22.3 | 7.9 | 0.5 | 10 | 44.9 | 10.73 | 56.85 | 10 | 42.92 | 88.8 | 138.58 | 0.0003 | [23] |
| *Ctenopharyngodon idellus* | 772.68 | 741.7728 | — | 21 | 7.9 | 1 | 10 | 44.9 | 10.73 | 54.55 | 10 | 42.92 | 88.8 | 138.58 | 0.0003 | [23] |
| *Ctenopharyngodon idellus* | 831.19 | 797.9424 | — | 21 | 7.9 | 0.05 | 10 | 44.9 | 10.73 | 52.25 | 10 | 42.92 | 88.8 | 138.58 | 0.0003 | [23] |

^a^ Values of HA in the column are not reported by the author, 10% of the default value is used.

^a^ Values of S^2-^ in the column are not reported by the author, 1×10^-10^ of the default value is used.

**References**

1. Schubauer-Berigan MK, Dierkes JR, Monson PD, Ankley. GT. pH-dependent toxicity of cadmium, copper, nickel, lead and zinc to *Ceriodaphnia dubia*, *Pimephales promelas*,*Hyalella azteca* and *Lumbriculus variegatus*. Environmental Toxicology and Chemestry. 1993;12(7):1261-6.

2. Carlson AR, Nelson H, Hammermeister D. Development and validation of site-specific water quality criteria for copper. Environmental Toxicology and Chemistry 1986;5(11):997-1012.

3. Belanger SE, Farris JL, Cherry DS. Effects of diet, water hardness, and population source on acute and chronic copper toxicity to Ceriodaphnia dubia. . Archieves of Environmental Contamination and Toxicology 1989;18(4):601-11.

4. Belanger SE, Cherry DS. Interacting effects of pH acclimation, pH, and heavy metals of acute and chronic toxicity to *Ceriodaphnia dubia* (Cladoceran). Journal of Crustacean Biology. 1990;10(2):225-35.

5. Oris JT, Winner RW, Moore MV. A four-day survival and reproduction toxicity test for *Ceriodaphnia dubia*. (2):. Environmental Toxicology and Chemistry 1991;10(2):217-24.

6. Diamond JM, Koplish DE, Iii MM, Rost R. Evaluation of the water-effect ratio procedure for metals in a riverine system. Environmental Toxicology & Chemistry. 1997;16(3):509-20.

7. Nebeker AV, Cairns MA, Onjukka ST, Titus RH. Effect of age on sensitivity of *Daphnia magna* to cadmium, copper and cyanazine. Environmental Toxicology & Chemistry. 1986;5(6):527-30.

8. Baird DJ, Barber I, Bradley M, Soares AM, Calow P. A comparative study of genotype sensitivity to acute toxic stress using clones of *Daphnia magna* straus. Ecotoxicology & Environmental Safety. 1991;21(3):257-65.

9. Meador JP. The interaction of pH, dissolved organic carbon, and total copper in the determination of ionic copper and toxicity. Aquatic Toxicology. 1991;19(1):13-31.

10. USEPA. Aquatic Life Ambient Freshwater Quality Criteria-Copper, EPA-822-R-07-001; United States Environmental Protection Agency Office of Water 4304T: Washington DC. 2007.

11. Zhou T, Cao Y, Qin L, Zhang Y, Zeng H, Yan Z, et al. Application of Biotic Ligand Model for the Acute Toxicity of Copper to *Daphnia magna* in water of Liao River and Taihu Lake. Environmental Science. 2014;35(5):332-7.

12. Buckley JA. Complexation of copper in the effluent of a sewage treatment plant and an estimate of its influence on toxicity to coho salmon. Water Research. 1983;17(12):1929-34.

13. Chapman GA. Toxicity of copper, cadmium and zinc to Pacific Northwest salmonids, U.S. EPA, Corvallis,OR. 1975.

14. Chapman GA, Stevens DG. Acutely lethal levels of cadmium, copper, and zinc to adult male coho salmon and steelhead. [Oncorhynchus kisutch; Salmo gairdneri]. Transactions of the American Fisheries Society. 1978;107:6.

15. Mudge JE, Northstrom TE, Jeane GS, Davis W, Hickam JL. Effect of varying environmental conditions on the toxicity of copper to salmon. In: Environmental toxicology and risk assessment. Gorsuch, J.W.,F.J. Dwyer, C.G. Ingersoll and T.W. LaPoint (Eds.). ASTM STP 1216. American Society for Testing and Materials, Philadelphia, PA. pp.19-33. 1993.

16. Welsh PG, Lipton J, Chapman GA, Podrabsky TL. Relative importance of calcium and magnesium in hardness-based modification of copper toxicity. Environmental Toxicology & Chemistry. 2000;19(6):1624-31.

17. Chapman GA. Toxicities of cadmium, copper, and zinc to four juvenile stages of chinook salmon and steelhead. [*Oncorhynchus tsawytscha*; *Salmo gairdneri*]. Transactions of the American Fisheries Society. 1978;107:6.

18. Chakoumakos C, Russo RC, Thurston RV. The toxicity of copper to cutthroat trout (*Salmo clarki*) under different conditions of alkalinity, pH, and hardness. Environmental Science ＆Technology. 1979;13:213-9.

19. Cusimano RF, Brakke DF, Chapman GA. Effects of pH on the Toxicities of Cadmium, Copper, and Zinc to Steelhead Trout ( Salmo gairdneri ). Canadian Journal of Fisheries & Aquatic Sciences. 1986;43(43):1497-503.

20. Lü YB, Li GG, Gong ZY, Wang Q. To predict copper toxicity in China 5 main rivers by using biotic ligand model (BLM). Acta Scientiae Circumstantiae. 2006;26(12):2080-5.

21. Cairns J, Jr., Thompson KW, Hendricks AC. Effects of fluctuating, sublethal applications of heavy metal solutions upon the gill ventilation response of bluegills (*Lepomis macrochirus*). EPA-600/3-81-003. National Technical Information Service, Springfield, VA. 1981.

22. Wang CY, Chen H, Zheng BH, An LH. Bioavailability of Cu to medaka in Xiangjiang with biotic ligand model. Asian Journal of Ecotoxicology. 2013;8(6):998-1004.

23. Wang W, Chen S, Wu M, Zhao J. Predicting Copper Toxicity to *Hypophthalmichthys molitrix* and *Ctenopharyngodon idellus* Based on Biotic Ligand Model. Environmental Sciences. 2014;35(10):3947-51.
